# Gambling disorder (GD) in youth with borderline personality disorder: Understanding comorbidity

**DOI:** 10.1192/j.eurpsy.2021.1988

**Published:** 2021-08-13

**Authors:** V. Kaleda, E. Krylova, A. Kuleshov, A. Beburishvili

**Affiliations:** 1 Department Of Youth Psychiatry, FSBSI «Mental Health Research Centre», Moscow, Russian Federation; 2 Department Of Youth Psychiatry, Mental Health Research Center, Moscow, Russian Federation; 3 Department Of Youth Psychiatry, FEDERAL STATE BUDGETARY SCIENTIFIC INSTITUTION MENTAL HEALTH RESEARCH CENTER, Moscow, Russian Federation; 4 Youth Psychiatry, Mental Health Research Cebter, Moscow, Russian Federation

**Keywords:** youth, Borderline personality disorder, Gambling, comorbidity

## Abstract

**Introduction:**

Epidemiological data suggest that in youth the prevalence of co-occurring borderline personality disorder (BPD) is particularly high in people with gambling disorder (GD).

**Objectives:**

The objective of this study was to investigate clinical presentations of GD in youth patients with BPD.

**Methods:**

Clinical psychopathological interview, SCID-II, The Gambling Symptom Assessment Scale (G-SAS), Hamilton Depression Rating Scale (HDRS), Zung Anxiety Rating Scale (ZARS). Sample: N=65 male and female, age: 18-25 with GD and BPD.

**Results:**

GD clinical presentation in BPD patients in youth have age and individual specific signs, like polymorphism and high conjugacy with comorbid mental disorders (including, but not limited to MDD, OCD, anxiety disorders, body dysmorphic disorder and etc.) Types of GD in BPD varied due to these comorbid syndrome: 1. Subjects with GD, BPD and MDD in youth demonstrated severity progression in anticipatory tension emotional distress (mental pain, shame, guilt) and lower level in pleasure on winning the bet (G-SAS:SD/Mean 35± 3). 2. Group with OCR and Anxiety Disorders showed different profile: urges to gamble and emotional distress dominated here (G-SAS SD/Mean 32.5± 1). 3. Individuals with no clear co-occurring clinical syndromes revealed combination low level control thoughts of gambling with much excitement and pleasure on winning the bet (G-SAS:SD/Mean 41.2± 2).

**Conclusions:**

Our research provides further insight on GD structure in youth BPD patients with comorbid psychiatric syndromes

**Disclosure:**

No significant relationships.

